# Menopausal hormone therapy in Türkiye: physician knowledge and practice gaps

**DOI:** 10.1590/1806-9282.20260307

**Published:** 2026-08-21

**Authors:** Mehdi Erdem, Nisa Akın Yetkin, Ali Can Güneş

**Affiliations:** 1 Sincan Training and Research Hospital, Department of Endocrinology and Metabolism – Ankara, Türkiye.; 2 Sincan Training and Research Hospital, Department of Internal Medicine – Ankara, Türkiye.; 3 Hacettepe University, Department of Obstetrics and Gynecology – Ankara, Türkiye.

**Keywords:** Menopause, Hormone replacement therapy, Physicians, Surveys and questionnaires

## Abstract

**OBJECTIVE:**

The aim of this study was to evaluate physician awareness, attitudes, and prescribing practices regarding menopausal hormone therapy in Türkiye and to identify specialty-related practice gaps in the context of evolving evidence.

**METHODS:**

A cross-sectional online survey was conducted between January and June 2025 among physicians in obstetrics and gynecology, endocrinology, and internal medicine. A 15-item structured questionnaire assessed menopausal hormone therapy knowledge, attitudes, and prescribing behaviors. Descriptive statistics and chi-square tests were used, with p<0.05 considered statistically significant. Multivariable logistic regression was performed to identify factors associated with willingness to initiate menopausal hormone therapy.

**RESULTS:**

A total of 235 physicians participated. Overall, 53.2% reported familiarity with menopausal hormone therapy guidelines, with significant differences across specialties (p<0.001); internal medicine physicians demonstrated the lowest awareness and the highest self-reported inadequate knowledge (94.4%). Willingness to prescribe menopausal hormone therapy for symptomatic women aged 50–59 years differed significantly by specialty (p<0.001), highest among obstetrics and gynecology physicians and lowest among internal medicine physicians. In adjusted analysis, internal medicine (aOR 0.179, 95%CI 0.067–0.474; p=0.001) and endocrinology (aOR 0.252, 95%CI 0.120–0.531; p<0.001) were independently associated with lower willingness to initiate menopausal hormone therapy.

**CONCLUSION:**

Significant specialty-dependent gaps in menopausal hormone therapy knowledge and prescribing confidence persist in Türkiye. Targeted education and improved dissemination of contemporary guidelines may help reduce undertreatment of symptomatic menopausal women.

## INTRODUCTION

Menopause is a universal biological transition characterized by declining ovarian estrogen production and cessation of reproductive capacity, typically occurring between 50 and 52 years of age in high-income countries, with earlier onset reported in some populations^1^. Estrogen deficiency affects multiple organ systems and may lead to vasomotor symptoms, sleep disturbance, mood changes, sexual dysfunction, genitourinary syndrome of menopause, and accelerated bone loss, all of which can significantly impair quality of life.

Menopausal hormone therapy (MHT) remains the most effective treatment for vasomotor symptoms and provides additional benefits for bone and genitourinary health^1–3^. However, global prescribing patterns changed dramatically after publication of the Women's Health Initiative (WHI) trials in 2002, which reported increased cardiovascular and breast cancer risks in older women initiating combined therapy many years after menopause^3,4^. These findings contributed to persistent therapeutic hesitancy among clinicians.

Subsequent analyses demonstrated that risks are strongly modified by age and time since menopause. Women aged 50–59 years or within 10 years of menopause generally derive symptom benefit and may experience net clinical advantage when appropriately selected^2,5^. Contemporary guidelines therefore recommend individualized risk–benefit assessment and shared decision-making.

Despite evolving evidence, important knowledge and confidence gaps remain among physicians, particularly outside obstetrics and gynecology. Data from Türkiye are limited. Therefore, this study aimed to evaluate awareness, attitudes, and prescribing behaviors related to MHT among physicians in endocrinology, internal medicine, and obstetrics and gynecology.

## METHODS

This cross-sectional, questionnaire-based study evaluated physicians’ awareness, attitudes, and prescribing behaviors regarding MHT in Türkiye. The survey was conducted online between January and June 2025 and targeted specialists in endocrinology, internal medicine, and obstetrics and gynecology. Participants were recruited through professional networks, institutional mailing lists, and online distribution platforms (including social media channels). Due to the distribution strategy, a precise response rate could not be calculated. Eligible participants were board-certified or actively practicing physicians in these specialties. Physicians in residency training were also eligible. Participation was voluntary and anonymous, and no financial incentives were provided.

The survey instrument, titled *Menopausal Hormone Therapy Awareness and Physician Approach Questionnaire*, consisted of 15 structured items developed based on current guideline recommendations (NAMS, Endocrine Society, ESHRE). The questionnaire covered three domains: demographics/professional background, knowledge and awareness of MHT, and prescribing attitudes/practices. The questionnaire was developed based on current guideline recommendations and reviewed by clinicians experienced in menopause management. Formal validation was not performed. Self-reported knowledge levels ("adequate" vs. "inadequate") were based on participants’ subjective assessment rather than predefined objective criteria.

Data were analyzed using IBM SPSS Statistics (IBM Corp., Armonk, NY, USA). Categorical variables were expressed as counts and percentages. Comparisons were performed using chi-square or Fisher's exact test where appropriate. A two-sided p<0.05 was considered statistically significant.

The study was conducted in accordance with the principles of the Declaration of Helsinki and the ethical standards of Türkiye. Ethical approval was obtained from the Ethics Committee of Sincan Training and Research Hospital (Approval No: BAEK-2025-26, January 2025). Participation was voluntary and anonymous, and completion of the survey was accepted as informed consent.

## RESULTS

A total of 235 physicians completed the survey. The demographic and professional characteristics of the study participants are summarized in Table 1. The most common specialty was obstetrics and gynecology (63.4%), followed by endocrinology (21.3%) and internal medicine (15.3%). Most espondents were aged 30–39 years (55.3%) and were women (63.4%). Regarding workplace setting, 61.7% practiced in state hospitals, 21.3% in university hospitals, and 17.0% in private institutions.

**Table 1:** Baseline characteristics of the study participants.

Variable	Category	n (%)
Specialty	Internal medicine	36 (15.3%)
	Endocrinology	50 (21.3%)
	Obstetrics and gynecology	149 (63.4%)
Gender	Male	86 (36.6%)
	Female	149 (63.4%)
Age group (years)	<30	24 (10.2%)
	30–39	130 (55.3%)
	40–49	32 (13.6%)
	≥50	49 (20.9%)
Years in practice	0–5	67 (28.5%)
	6–10	76 (32.3%)
	11–20	36 (15.3%)
	>20	56 (23.8%)
Institution type	University hospital	50 (21.3%)
	State hospital/training and research hospital	145 (61.7%)
	Private hospital/clinic	40 (17.0%)

Values are presented as n (%). Percentages may not sum to 100 due to rounding. Years in practice refer to years since initiation of independent clinical practice after specialty training/certification (as self-reported). Institution type reflects the participants’ primary workplace at the time of survey completion. Training and research hospitals were grouped with state hospitals for analysis/presentation.

### Knowledge and awareness

The detailed findings regarding physicians’ knowledge and awareness of MHT are presented in Table 2. Overall, 53.2% of physicians reported familiarity with current MHT guidelines. Guideline familiarity significantly differed by specialty (p<0.001), with gynecologists (65.1%) and endocrinologists (48.0%) being more familiar compared to internal medicine physicians (11.1%). Most respondents correctly identified MHT indications (93.6%), although internal medicine physicians showed lower awareness (64.1%) relative to endocrinologists (96.0%) and gynecologists (100%) (p<0.001). Awareness of delivery routes varied significantly by specialty. Gynecologists demonstrated the highest awareness of vaginal, transdermal, and combined formulations (all p<0.001). In contrast, internal medicine physicians showed limited awareness of non-oral preparations (e.g., vaginal: 41.7%, transdermal: 44.4%).

**Table 2:** Menopausal hormone therapy knowledge and awareness by specialty.

Variable		Internal med (n=36)	Endocrinology (n=50)	Obstetrics and gynecology (n=149)	p-value
Familiarity with current MHT clinical guidelines		4 (11.1%)	24 (48.0%)	97 (65.1%)	**<0.001**
Knows clinical indications for MHT		23 (64.1%)	48 (96.0%)	149 (100%)	**<0.001**
Formulation awareness	Oral formulation	32 (88.9%)	40 (80.0%)	131 (87.9%)	0.329
	Vaginal formulation	15 (41.7%)	35 (70.0%)	130 (87.2%)	**<0.001**
	Transdermal formulation	16 (44.4%)	41 (82.0%)	134 (89.9%)	**<0.001**
	Combined	24 (66.7%)	39 (78.0%)	127 (85.2%)	0.034
Benefits endorsed	Osteoporosis	30 (83.3%)	45 (90.0%)	136 (91.3%)	0.368
	Vasomotor	34 (94.4%)	45 (90.0%)	139 (93.3%)	0.676
	Genitourinary	30 (83.3%)	43 (86.0%)	135 (90.6%)	0.387
	Cardiovascular	29 (80.6%)	43 (86.0%)	132 (88.6%)	0.434
Risks endorsed	Endometrial hyperplasia	28 (77.8%)	43 (86.0%)	118 (79.2%)	0.524
	Breast cancer	28 (77.8%)	44 (88.0%)	128 (85.9%)	0.381
	Thromboembolism	29 (80.6%)	48 (96.0%)	138 (92.6%)	**0.029**
Self-rated knowledge—adequate		0 (0.0%)	5 (10.0%)	44 (29.5%)	**<0.001**

Survey-derived measures of MHT-related knowledge and awareness by specialty (internal medicine, endocrinology, obstetrics & gynecology). Values are presented as n (%). p-values were calculated using Pearson's chi-square test; Fisher's exact test was used when expected cell counts were <5. A two-sided p-value <0.05 was considered statistically significant. Key statistically significant differences are highlighted within the table. MHT: menopausal hormone therapy. The bold values indicate statistically significant results (p<0.05).

The majority of physicians recognized major MHT benefits, including reductions in vasomotor symptoms (92.8%), osteoporosis (89.8%), and genitourinary symptoms (88.5%), with no significant specialty differences (all p>0.05). Most physicians endorsed cardiovascular protection as a benefit (86.8%), with no significant differences across specialties (p=0.434). Regarding perceived risks, most respondents identified breast cancer (85.1%), thromboembolism (91.5%), and endometrial hyperplasia (80.4%) as potential adverse effects. Awareness of thromboembolic risk differed by specialty (p=0.029), being highest among endocrinologists (96%) and lowest in internal medicine physicians (80.5%).

### Attitudes and prescribing practices

When asked whether they would initiate MHT in symptomatic women aged 50–59 years, responses significantly differed by specialty (p<0.001). Gynecologists were most likely to prescribe (58.4%), endocrinologists demonstrated a balanced pattern (32.0% yes, 50.0% no), and internal medicine physicians showed the highest reluctance (41.7% no; 38.9% unsure). Among respondents who would not prescribe MHT, reasons varied by specialty (p<0.001). Internal medicine physicians most frequently cited lack of knowledge (72.2%), whereas gynecologists cited cancer (58.9%) and thrombosis concerns (28.2%).

Preferred MHT routes differed markedly (p<0.001). Gynecologists favored transdermal (38.3%) and oral (27.5%) routes, endocrinologists exhibited mixed preferences, while internal medicine physicians most often selected no route (80.6%), reflecting low prescribing familiarity.

In terms of decision support, gynecologists and endocrinologists relied primarily on clinical guidelines, whereas internal medicine physicians more frequently relied on consultation and had the highest proportion reporting no decision source (p<0.001).

Self-reported knowledge differed significantly across specialties (p<0.001). "Adequate" knowledge was highest among gynecologists (29.5%), followed by endocrinologists (10%), and was absent among internal medicine physicians (0%). Conversely, 94.4% of internal medicine physicians rated their knowledge as "inadequate."

In multivariable logistic regression, specialty remained independently associated with willingness to initiate MHT. Compared with obstetrics and gynecology physicians, internal medicine physicians had markedly lower odds (aOR 0.179, 95%CI 0.067–0.474; p=0.001), and endocrinologists also had lower odds (aOR 0.252, 95%CI 0.120–0.531; p<0.001), whereas age, years in practice, institution type, and gender were not statistically significant predictors (Table 3).

**Table 3:** Factors associated with willingness to initiate menopausal hormone therapy in symptomatic women aged 50–59 years (multivariable logistic regression).

Predictor		Adjusted OR	95%CI	p-value
Specialty (ref: obstetrics & gynecology)				
	Internal medicine vs. OBGYN	0.179	0.067–0.474	**0.001**
	Endocrinology vs. OBGYN	0.252	0.120–0.531	**<0.001**
Age group (ref: >50)				
	<30 vs. >50	1.685	0.102–27.884	0.716
	30–39 vs. >50	4.786	0.355–64.511	0.238
	40–49 vs. >50	5.255	0.521–52.988	0.159
Years in practice (ref: >20**)**				
	0–5 vs. >20	0.171	0.012–2.389	0.189
	6–10 vs. >20	0.263	0.020–3.458	0.310
	11–20 vs. >20	0.180	0.016–2.025	0.165
Institution type (ref: private hospital/clinic)				
	University vs. private	0.785	0.283–2.183	0.643
	State vs. private	0.476	0.197–1.147	0.098
Gender (ref: female)				
	Male vs. female	1.360	0.746–2.481	0.315

Multivariable binary logistic regression examining factors associated with willingness to initiate MHT in symptomatic women aged 50–59 years (Yes vs. No/Undecided). Values are adjusted odds ratios (aOR) with 95% confidence intervals (CI). The model included specialty, age group, years in practice, institution type, and gender. Omnibus test: χ^2^(11)=42.474, p<0.001; Nagelkerke R^2^=0.221. CI: confidence interval; OR: odds ratio; OBGYN: Obstetrics and Gynecology. The bold values indicate statistically significant results (p<0.05).

## DISCUSSION

In this nationwide cross-sectional survey, we identified substantial specialty-dependent differences in knowledge, awareness, and prescribing practices related to MHT in Türkiye. Although most physicians recognized the principal benefits of MHT for vasomotor symptoms, bone protection, and genitourinary syndrome of menopause, only about half reported familiarity with contemporary clinical guidelines, a finding that is consistent with previous studies demonstrating persistent educational gaps in menopause management among physicians^6^. Importantly, willingness to initiate MHT in symptomatic women aged 50–59 years varied markedly by specialty, with the greatest reluctance observed among internal medicine physicians. These findings highlight persistent practice gaps that may contribute to undertreatment of symptomatic menopausal women. Notably, a high proportion of physicians also endorsed cardiovascular protection as a benefit of MHT. However, cardiovascular effects are context-dependent and influenced by timing of initiation, age, and baseline risk profile, rather than representing a universally protective effect.

The enduring impact of the WHI publications continues to shape clinician perceptions. Early interpretations of WHI emphasized increased cardiovascular and breast cancer risks, leading to a global decline in MHT use and long-lasting therapeutic caution. Subsequent analyses of the WHI trials have provided a more nuanced understanding of breast cancer risk according to hormone regimen and duration of therapy, helping to refine contemporary clinical recommendations^2,5,7^. Contemporary position statements from major societies now emphasize individualized assessment and shared decision-making rather than blanket avoidance^1,3,8^. Despite this paradigm shift, our findings suggest that many clinicians in Türkiye—particularly outside obstetrics and gynecology—remain anchored to earlier, more restrictive risk perceptions.

Barriers to prescribing differed meaningfully across specialties. Among gynecologists, concern regarding breast cancer predominated, whereas lack of knowledge was the principal barrier among internal medicine physicians. Similar patterns have been reported internationally, where persistent misconceptions regarding estrogen safety and inadequate menopause-related education contribute to therapeutic hesitancy^9–12^. These findings suggest that the knowledge and practice gaps identified in our study are unlikely to be unique to Türkiye, but rather reflect a broader global challenge in menopause care. Recent qualitative and survey-based studies further highlight substantial knowledge gaps, fears, and reliance on healthcare provider guidance among women navigating menopause^13^. In addition, digital health interventions such as structured educational platforms have shown promise in improving menopause literacy and patient engagement^14^.

Another clinically important observation was the substantial variation in awareness of MHT formulations. Gynecologists demonstrated the greatest familiarity with transdermal and vaginal preparations, whereas internal medicine physicians showed limited awareness of non-oral options. This gap has practical implications. Transdermal estradiol avoids first-pass hepatic metabolism and is associated with a lower risk of venous thromboembolism compared with oral therapy in observational studies^15–17^. Current guidelines, therefore, recommend considering transdermal routes in women with elevated cardiometabolic or thrombotic risk^3,15^. Improving physician familiarity with route-specific risk profiles may represent a modifiable target to optimize menopause care.

The intersection between menopause management and oncology further complicates clinical decision-making, particularly in women with gynecologic malignancies, for whom individualized assessment is essential^18^. Evidence indicates that short-term MHT may be acceptable in selected high-risk populations, such as BRCA mutation carriers undergoing risk-reducing salpingo-oophorectomy, without significantly increasing breast cancer risk^19–21^. Conversely, systemic MHT remains generally contraindicated in survivors of estrogen receptor–positive breast cancer, and management should be individualized using evidence-based hormonal and nonhormonal strategies when appropriate^22^. Likewise, women with a history of gynecologic malignancy require individualized consideration of menopausal hormone therapy according to cancer type, recurrence risk, and current evidence^23^. Although oncologic scenarios were not explored in detail in our survey, the high prevalence of generalized cancer concern suggests that many clinicians may lack confidence in nuanced risk stratification.

Nonhormonal therapies constitute important alternatives for women who cannot or prefer not to use MHT. Recent evidence supports the efficacy of SSRIs, SNRIs, gabapentin, and neurokinin-3 receptor antagonists such as fezolinetant for vasomotor symptom relief^24–26,8^. However, our survey did not assess familiarity with these agents. Future research should determine whether avoidance of MHT leads to appropriate evidence-based nonhormonal prescribing or to reliance on complementary therapies, including plant-based interventions, for which evidence of efficacy remains limited or variable^27^. Importantly, reduced use of MHT does not necessarily translate into appropriate use of evidence-based nonhormonal therapies and may instead reflect a broader pattern of undertreatment, particularly when both hormonal and nonhormonal options are underutilized.

This study has several limitations. Its cross-sectional, online design may introduce selection bias and rely on self-reported practices rather than objectively verified prescribing behavior. Detailed oncologic and high-risk clinical scenarios were not assessed, and familiarity with nonhormonal treatment options was not captured. In addition, the sample was limited to Türkiye and may not fully reflect international practice patterns. Furthermore, the questionnaire was not formally validated.

Despite these limitations, our findings provide important real-world insight into menopause care across specialties. Targeted, multidisciplinary educational initiatives and wider dissemination of updated guideline recommendations appear warranted to improve physician confidence and ensure that symptomatic women receive individualized, evidence-based counseling, particularly for internal medicine and endocrinology physicians, who play a key role in managing midlife women but may have limited formal training in menopause care. Potential strategies include integrating menopause-focused education into internal medicine and endocrinology training, promoting multidisciplinary collaboration, and improving accessibility of up-to-date clinical guidelines. From a public health perspective, structured national policies and comprehensive women's health programs may further influence menopause-related outcomes, as suggested by population-level analyses^28^.

## CONCLUSION

This study underscores the ongoing unmet needs of menopausal women, many of whom experience substantial symptom burden yet receive limited counseling or treatment due to physician hesitancy, outdated risk perceptions, or lack of menopause training. Closing this gap requires improving physician familiarity with current evidence and guidelines, expanding access to both hormonal and nonhormonal therapies, and supporting shared decision-making. Strengthening physician education in menopause management has the potential to improve quality of life, long-term health outcomes, and overall satisfaction with care among midlife women.

## Data Availability

The datasets generated and/or analyzed during the current study are available from the corresponding author upon reasonable request.
